# Green and red macroalgae extracts show antibacterial effects and induce innate immune responses in Nile tilapia and rainbow trout *in vitro*

**DOI:** 10.1016/j.cirep.2023.200128

**Published:** 2023-12-22

**Authors:** Jules Petit, Erik van den Brink, Pi Nyvall Collén, Olga L.M. Haenen, Johan Schrama, Geert F. Wiegertjes

**Affiliations:** a Aquaculture and Fisheries Group, Department of Animal Sciences, Wageningen University & Research, Wageningen, the Netherlands; b OLMIX SA, ZA du Haut du Bois, 56 580, Bréhan, France; c National Reference Laboratory for Fish Diseases, Wageningen Bioveterinary Research, Wageningen University and Research, Lelystad, the Netherlands

**Keywords:** Immunomodulation, Macroalgae, Marine sulphated polysaccharides, Ulva, Soleria

## Abstract

Green macroalgae (*Ulva* sp.) and red macroalgae (*Solieria* sp.) were processed to produce eight different crude extracts enriched for marine sulphated polysaccharides (MSP). While all extracts showed direct anti-bacterial effects to some degree, two red algal extracts, in particular, showed high activity against several pathogenic fish bacteria *in vitro*. Stimulation of head kidney leukocytes (HKLs) *in vitro* with MSP-rich extracts showed fish species specific differences. In Nile tilapia, HKLs showed a dose dependent production of reactive oxygen species (ROS) following stimulation with *Ulva-*derived extracts, while stimulation with *Solieria*-derived extracts did not induce ROS production. In rainbow trout, HKLs showed relatively high reactive ROS potential and *Solieria*-derived extracts could induce significant ROS production, albeit without a clear dose dependent response. Gene expression of *in vitro* stimulated HKLs showed a clear induction of most cytokines measured (*il1b, il10, tnfa, ifny, il12p40*), with differences between species. Although cytokine gene expressions were more prominently induced in HKLs of Nile tilapia than in HKLs of rainbow trout, immunomodulatory effects of both, *Ulva-* and *Solieria-*derived extracts, always appeared evident. It is likely that HKLs have the ability to sense presence of MSP, via so far uncharacterized pattern recognition receptors which have known downstream signalling pathways. Expression of some genes associated with Toll-like Receptor (TLR) signalling, such as *irak1* and especially the expression of genes associated with C-type Lectin Receptor (CLR) signalling, such as *card9* and *bcl10* were evident following stimulation with MSP-rich extracts. Possibly, immunomodulatory effects of MSP could be regulated by at least a CLR-mediated signalling, at least in Nile tilapia, for which most if not all red algae and green algae extracts induced changes in gene expression of *card9* and *bcl10*. Overall, induction of ROS production and gene expression read-outs suggest immunomodulatory effects of MSP-rich extracts derived from green algae (*Ulva* sp.) and red algae (*Solieria* sp.), at least *in vitro*. The observed effects suggest clear fish species specific differences between the effects of MSP-rich extracts.

## Highlights

• Marine sulphated polysaccharides (MSP) have direct antibacterial effects *in vitro*.
• The antibacterial effect of MSP is dependent on seaweed species and extraction.
• MSP-rich extracts modulate fish immune responses.
• Effects of MSP-rich extracts on head kidney leukocytes appear fish species specific.
• Differences exist between seaweed species in MSP-driven immune modulation.

## Introduction

To keep up with an increasing demand for animal protein, the aquaculture sector has seen an increase in production over the past decades, among others due to intensification of the sector. Intensification of the aquaculture sector has also been accompanied by an increase in disease incidences. Besides prevention of diseases through vaccination, and treatment of diseases by antibiotics, dietary supplementation with immunomodulators may provide an alternative route to maintain animal health in aquaculture. Among the better-known immunomodulatory feed additives are non-starch polysaccharides, with β-glucans probably being the best characterized compounds [as reviewed by: [1,2]]. Another group of polysaccharides with potentially immunomodulatory effects are marine sulphated polysaccharides (MSPs) isolated from macroalgae. While MSP-rich extracts containing health-promoting compounds are gaining interest from the aquaculture sector, their immunomodulatory effects are not always clearly defined.

Cell wall matrices of marine algae commonly contain highly complex sulphated polysaccharides. Polysaccharides extracted from green macroalgae are referred to as ulvans, while fucoidans are extracted from brown algae and carrageenans from red algae. Several studies have shown such polysaccharide compounds to exhibit a wide range of biological activities, including immunomodulatory properties, mostly in mammalian studies (Reviewed in: [3]). Desulphation is generally accepted to decrease their bioactivity, indicating sulphate residues are important for their activity [4]. Among the better studied are the ulvans; water-soluble sulphated polysaccharides composed of disaccharide repetition moieties made up of sulphated rhamnose linked to either glucuronic acid, iduronic acid or xylose. Stimulation of immune cells with ulvans has been shown to trigger events leading to the activation of the immune system or the control of the inflammation process [5,6]. Mammalian literature shows that ulvans are able to enhance macrophage functions such as phagocytosis and induce production of reactive oxygen species (ROS) and nitric oxide (NO), and lead to the secretion of pro-inflammatory cytokines such as tumour necrosis factor (TNFα), interleukins IL-1, IL-6, IL-8, IL-12, and interferon (IFN) [[7], [8], [9], [10]]. Interestingly, incubation of murine (RAW) macrophages with *U. rigida* polysaccharides induced an increase in nitrite production which could be decreased considerably by desulphation, arguing for an important role of the sulphate groups in the induction of this immune activation by *U. rigida* derived polysaccharides [11]. Further, ulvans, carrageenans and fucoidans have been shown to regulate innate immune responses through Toll-like receptors such as TLR4 and TLR2 expressed on target cells, inducing signalling pathways that lead to the activation of the transcription factor NF-kappa B [[12], [13], [14], [15], [16]]. Taken together, immunomodulatory effects of marine sulphated polysaccharides, or MSP, on mammalian and chicken cells, at least *in vitro*, are well established.

Previously, the Olmix Group investigated the potential of marine macroalgae harvested on the shore of France to modulate the immune response of porcine intestinal epithelial cells (IPEC-1), or human embryonic kidney cells (HEK293) [12,17]. Aqueous MSP-rich extracts prepared from *Ulva* sp. were evaluated for antibacterial and immunomodulatory activities *in vitro*. MSP-rich extracts were shown to directly inhibit the growth of pathogenic bacteria and stimulate gene expression of the cytokines IL-1, IL-6, IL-8, TNFα, TGFβ and chemokine CCL20 [17]. Further, MSP-rich extracts were used to shed light on the molecular mechanisms underlying their biological activity, evaluating a panel of pattern recognition receptors expressed on HEK cells to identify the target receptor and downstream cytokine expression. MSP-rich extracts prepared from *Ulva* sp. primarily stimulated TLR4, and Western blot analyses of MSP-treated HEK293-TLR4 cells showed an increase in the phosphorylation of Akt and the p65 subunit of nuclear factor-κB. Inhibition of Akt phosphorylation with a specific inhibitor abrogated MSP-mediated enhancement of IL-8 secretion [12]. It was thus concluded that MSP-rich extracts prepared from *Ulva* sp. could represent TLR ligands with immunomodulatory properties. It is important to take a comparable approach to the understanding of the action and mechanisms of MSP-mediated immunomodulatory activities in fish cells prior to their use as health-improving compounds for future preventive strategies in aquaculture.

For aquaculture, application of fucoidans as immunomodulating compounds has recently been reviewed, including *in vivo* effects on growth, intestinal health, antioxidant capacity, and immune responses of several finfish and shellfish species [18]. However up to date, there are not many *in vitro* studies in fish that aim to provide detailed information on potential working mechanisms of MSPs. In early studies with turbot (*Psetta maxima*), protein-free water-soluble extracts and polysaccharide fractions obtained from the green seaweed *Ulva rigida* and the red seaweed *Chondrus crispus*, these extracts could induce an increase in the respiratory burst activity of turbot phagocytes [19]. Stimulatory capacities of the different extracts varied depending on origin, concentration and time of incubation. In a follow-up study, the authors separated water-soluble *U. rigida* polysaccharides into uronic acids, rhamnose and xylose and found that especially, the acid sugar fractions with large homogeneous sulphated polysaccharides induced respiratory burst activity [20]. Of interest, this capacity was lost after desulphation, while recovered after non-specific resulphation, suggesting an important function of the sulphate groups for immunomodulation [20]. More recently, sulphated polysaccharides from *Codium fragile* (sponge seaweed) were shown to exhibit not only direct anti-bacterial activities against specific fish pathogenic bacteria but also to have immunostimulatory effects *in vitro* on head kidney and spleen leukocytes from rockfish (*Sebastes schlegelii*), using induction of gene expression of the cytokines *tnfα, il1b, il6, il8* and *il10* as read-out [21]. Together these studies suggest it could be informative to perform *in vitro* studies on the effects of MSP-rich extracts on cell-based mechanisms determining innate immune activity, in different fish species*.*

In the current study, we first investigated the effects of different crude extracts from green macroalgae (*Ulva* sp.) or red macroalgae (*Solieria* sp.) rich in MSPs for direct antibacterial effects *in vitro*. Further, head kidney leukocytes (HKL) isolated from Nile tilapia (*Oreochromis niloticus*) and from rainbow trout (*Oncorhynchus mykiss*) were stimulated with different MSP-rich extracts, using induction of reactive oxygen species (ROS) and gene expression as immunomodulatory read-outs. We discuss the results with regard to differences related to the source of MSP (*Ulva* or *Solieria* macroalgae), extraction method, suitability of immunomodulatory read-outs (ROS, gene expression), possible cellular signalling pathway (TLR, CLR) and fish species (Nile tilapia and rainbow trout).

## Material and methods

### Production of MSP-rich algae extracts

Green tide algae *Ulva* sp. were collected on beaches in Brittany, France at Plestin les Grèves (48° 39′ 28′′ N, 3° 37′ 47′′ W) and Guisseny (48°38′22’’N 4°26′47’’W). The biomass of the bulk samples analysed contained a mixture of two foliose Ulva species, predominantly *Ulva lacinulata* (Kützing) (previously known as *U. laetevirens* (Areshoug) or *U. armoricana*; (Dion, Reviers & Coat)), [22], and a lower amount of *Ulva* sp. A (previously known as *Ulva rigida*; [23]. Solier's red string weed (*Solieria chordalis;* [24] was collected on a beach in Vendée, France at Saint-Hilaire de Riez (46°42′52’’N 1°58′46’’W). The algae were washed in fresh water, drained, and deep frozen. For extraction, the algae were thawed; wet ground, and liquid and solid phases were separated by band press as part of an industrial process (Patent No. FR 61909). The liquid was fractionated by tangential filtration (Tami Industries). The solid fractions were further processed using different methods (Table 1).

**Table 1:** Description of different processing methods for green and red algae extracts. MSP-rich extracts are divided by species and by processing method.

Species	Processing method	Extract abbreviation
*Ulva* sp.	No extra processing	UC (Ulva concentrate)
	2 h of thermal processing at 80 °C	UC-T (-thermal)
	Protease assisted extraction	UC-E (-extracted)
*Solieria chordalis*	No extra processing	SC (Solieria concentrate)
	2 h of thermal processing at 80 °C	SC-T (-thermal)
	Protease assisted extraction	SC-E (-extracted)
*Ulva* sp.	Filtrate of a 15 kDa filtration	UC-F15 (-filtration)
	Filtrate of a 50 kDa filtration	UC-F50 (-filtration)

The percentage elemental analysis (C, N, S) elementar Analysensysteme GmbH-vario EL III Element Analyzer (Hanau, Germany) was applied according to the manufacturer's recommendations. Neutral sugars were determined using the phenol sulfuric acid method with anhydrous d-glucose used as standard (0–100 μg mL^−1^) [25]). Uronic acids were determined using the meta-hydroxydiphenyl method [26]. The amount of sulfate groups present on the polysaccharides were determined by the Azure A method (Sigma-Aldrich, France) using 17 % of sulfated dextran as standard in the range of 0 to 100 μg mL^−1^ [27]. The molar ratio of monosaccharides was determined after acidic methanolysis of the MSP sample using 3 mL^−1^ of MeOH/HCl for 4 h at 100 °C, followed by gas chromatography (GC) analysis of the trimethylsilyl derivatives using the method of Montreuil et al. [28]. Myoinositol (1 mg mL^−1^) was used as the internal reference, nitrogen as a carrier gas and a non-polar column, type HP-5MS (30 m, 0.25 mm internal diameter) for analysis. The temperature profile was: 120 °C for 1 min, followed by a gradient of 1.5 °C min^−1^ between 120 and 180 °C and 2 °C min^−1^ between 180 and 200 °C. Because of the high content of salt in the sample interfering with the reproducibility of the analyses, salts were eliminated by ultra-filtration using a 1-kDa cut-off Pall minimate membrane (Pall Corporation, France) and freeze-dried. Protein content was quantified by the bicinchoninic acid colorimetric method (BCA) using the Micro BC Assay Kit (Interchim, France) [29]. The molecular weight distribution of the sample (0.5 mg mL^−1^) was analyzed by size exclusion chromatography in 0.1 M sodium nitrate with 0.2 % sodium azide at a flow rate of 0.5 mL min^−1^. The Shodex 802 and 803 columns in series and a multi-angle light scattering refractometer (Wyatt, 18 angles) for detection were used with a dn/dc of 0.150 mL g^−1^. The different algal extracts were dissolved under sterile conditions by gentle agitation for 2 h at 37 °C in D-PBS (Gibco) and pasteurized for 20 min at 80 °C and stored at 4 °C until used.

### Antibacterial effects of MSP-rich algae extracts

Antimicrobial activity of the MSP-rich extracts was investigated against 13 fish bacterial strains (Table 2). Bacterial strains were identified by and obtained from the National Reference Laboratory for Antimicrobial Resistance and for Fish Diseases, Lelystad, the Netherlands (Wageningen Bioveterinary Research, WBVR). Bacteria were cultured overnight at 22 °C on heart infusion agar with 5 % sheep blood (HIS)-plates (*Aeromonas hydrophila, Edwardsiella tarda, Photobactrium damselae, Aeromonas salmonicida salmonicida, Edwardsiella ictaluri, Vibrio anguillarum, Yersina ruckeri, Aeromonas veronii, Shewanella putrefaciens* and *Vibrio vulnificus*), for four days at 22 °C on HIS-plates (*Streptococcus iniae* and *Streptococcus agalactiae*) or for four days at 15 °C on Marine agar plates (*Aliivibrio salmonicida*). Subsequently, individual colonies of each strain were suspended in sterile physiological saline solution (sodium chloride 0.85 %) and bacterial suspension density was adjusted to equal to 0.5 McFarland standard. Next, concentration of suspension equal to 0.5 McFarland was determined per strain by performing a ten-times dilution series (10^−1^ – 10^−10^) in triplicate. Dilution was performed on HIS-plates and cultured overnight at 22 °C (*A. hydrophila, E. tarda, P. damselae, A. salmonicida salmonicida, E. ictaluri, V. anguillarum, Y. ruckeri, A. veronii, S. putrefaciens* and *V. vulnificus*), on HIS-plates and cultured for four days at 22 °C (*S. iniae* and *S. agalactiae*) or on Marine agar plates and cultured for four days at 15 °C (*Aliivibrio salmonicida*). Based on the CFU mL^-1^ as determined by titration, 0.5 MacFarland was prepared in 9 ml sterile physiological saline solution and subsequently diluted to 10^5^ CFU mL^−1^ in Mueller Hinton Broth (MH broth). All eight different MSP-rich extracts (see Table 1) were first dissolved in ultra-pure water and sterilized by autoclaving, subsequently a two-times serial dilution was made from 25 mg mL^−1^ – 0.012 mg mL^−1^ in MH broth. In a sterile culture plate, 100 µL bacterial suspension (equal to 10^4^ CFU) was combined with 100 µL MSP solution dissolved in MH broth, resulting in a two-times serial dilution series from 12.5 mg mL^−1^ - 0.006 mg mL^−1^. Each plate included a positive control with bacterial strain alone in MH broth, and two negative controls; only MSP rich extract at the different dilutions, and only MH broth. For each bacterial strain, each concentration of MSP-rich extract was tested in three independent culture plates. For both *Streptococcus* strains, MH broth was supplemented with 5 % defibrinated sheep blood. Bacteria were incubated for 144 h at 22 °C, except *Aliivibrio salmonicida* which was incubated for 144 h at 15 °C. Following 24 h, 48 h, 72 h and 144 h incubation, or 72 h, 96, 120 h and 144 h for slow growing bacteria (*i.e. Aeromonas salmonicida, Aliivibrio salmonicida, Streptococcus agalactiae, Streptococcus iniae*) optical density was measured at O.D. 600 nm and plates were photographed. Antimicrobial effects were determined as the lowest MSP extract concentration required to inhibit growth (O.D. 600 nm with visually confirmation from photographs) of tested microorganisms.

**Table 2:** Bacterial strains used for analysis of direct antimicrobial effects of MSP-rich extracts.

Bacterial strains	Strain reference	Remarks
*Aeromonas hydrophila*	V82-4 311184	Gram-negative
*Edwardsiella tarda*	Norway 135.3195	Gram-negative
*Edwardsiella ictaluri*	USA A1-95-58	Gram-negative
*V. anguillarum*	ATCC 19564	Gram-negative
*Yersinia ruckeri*	66367	Gram-negative
*Aeromonas veronii*	13007418-2-1	Gram-negative
*Shewanella putrefaciens*	13007465-3	Gram-negative
*Vibrio vulnificus*	7025836	Gram-negative
*Photobacterium damselae*	10002263-1	Gram-negative
*Aeromonas salmonicida salmonicida*	ATCC 33658	Gram-negative
*Aliivibrio salmonicida*	DSMZ 21110	Gram-negative
*Streptococcus agalactiae*	5004591	Gram-positive
*Streptococcus iniae*	90041881-2A	Gram-positive

### Animals

Nile tilapia (*Oreochromis niloticus*) were reared at 28 ± 2 °C temperature with a 12–12 h light-dark cycle in the Aquatic Research Facilities of Wageningen University and Research (Carus-ARF, Wageningen, The Netherlands). Fish were fed a commercial diet twice per day (Sparos, Portugal). Rainbow trout (*Oncorhynchus mykiss*) were reared at 14 ± 0.5 °C temperature with a 12–12 h light-dark cycle, also in Carus-ARF. Fish were fed a trout specific research diet twice per day (Research Diet Services B.V., The Netherlands). Both Nile tilapia and rainbow trout were housed under comparable conditions, *n* = 30 fish per 200 L rectangular tank. Fish were 6 – 9 months old during the period that the animals were used. Tanks were connected to a recirculation system, including a trickling filter, sump, drum filter (Hydrotech 500®) and oxygenation unit. The flow-through for each tank was set at 7 L/min using a hand held liquid rotameter. Water quality parameters were checked regularly to ensure that the water quality remained within pre-set ranges. During the experiment with tilapia, ranges were for temperature 27.8–28.2 °C, pH 7.0–7.8, dissolved oxygen 4.7 and 7.5 mg/L, total ammonia nitrogen < 5 mg/L, nitrite < 1 mg/L and nitrate < 500 mg/L, all within the pre-set ranges. During the experiment with rainbow trout, ranges were for temperature 14.6–16.2 °C, pH 6.9–8.0, dissolved oxygen 4.7 and 7.5 mg/L, total ammonia nitrogen < 5 mg/L, nitrite < 1 mg/L and nitrate < 500 mg/L, all within the pre-set ranges. All experiments were performed with a permit (DEC number 2017.W-0034) approved by the Central Committee for Animal Testing, and supervised by the Animal Welfare Authority of Wageningen University and Research.

### Head kidney leukocyte (HKL) isolation

Fish were euthanized with 0.3 g L^−1^ tricaine methanesulphonate in aquarium water buffered with 0.6 g L^−1^ sodium bicarbonate (Nile tilapia) or 2-phenoxyethanol (1 mL L^−1^) in aquarium water (rainbow trout), and then bled via the caudal vein. Head kidneys were removed aseptically and total head kidney leukocytes (HKLs) were immediately separated on a Percoll (GE Healthcare, Thermo Fisher Scientific) density gradient (51 %), as previously described for Nile tilapia [30], and for rainbow trout [31]. Directly following isolation, HKLs were resuspended in RPMI-1640 (Gibco) supplemented with 100 IU penicillin/streptomycin (p/s) without serum (in case of ROS analysis) or supplemented with 1.5 % pooled tilapia or trout serum (depending on species). Cells were directly used after isolation in reactive oxygen or gene expression experiments.

### Induction of reactive oxygen species (ROS) production by MSP extracts

Production of ROS was determined by a real-time luminol-based luminescence assay, as previously described [32], with minor modifications. Briefly, HKLs were seeded in RPMI-1640 (Gibco) supplemented with 100 IU p/s at a density of 1 × 10^6^ per well and incubated for 60 min at 27 °C (Nile tilapia) or at 19 °C (rainbow trout) in white 96-well plates (CLS3912; Corning). Subsequently, cells were stimulated with one of the following: RPMI-1640 cell culture medium (negative control), zymosan (positive control; tlrl-zyd, 50 µg mL^−1^, InvivoGen) or one of the MSP-rich extracts (Table 1) at concentrations of 250, 500, 750, 1000 and 1500 µg mL^−1^. Per independent experiment, all stimuli were performed on HKLs isolated from the same animal. For each independent experiment, each condition was tested in triplicate. For ROS analysis, *n* = 4 independent experiments (*n* = 4 fish) per species was performed. Chemiluminescence emission was measured in real time (every 2 min for 120 min) with a FilterMax F5 Multi-Mode Microplate Reader at 27°C (Nile tilapia) or at 19 °C (rainbow trout), and expressed as fold changes based on areas under the curve [33]. Fold changes were based on data from stimulated relative to unstimulated HKLs (treated with RPMI).

### Head kidney leukocyte stimulation for gene expression analysis

For gene expression analysis, HKLs were seeded in RPMI-1640 (Gibco), supplemented with 100IU p/s and 1.5 % pooled tilapia or trout serum, at a density of 4.5 × 10^6^ per well in 24-well plates (CORN3524; Corning) and stimulated with RPMI (negative control), zymosan (tlrl-zyd, 50 mg ml^−1^, InvivoGen) or one of the extracts (Table 1) at a concentration of 500 µg mL^−1^ and incubated at 27 °C (Nile tilapia) or at 19 °C (rainbow trout). At 3 and 6 h post stimulation, cells were lysed in RLT lysis buffer (Qiagen, 79,216) and stored at −80°C until RNA isolation. Per independent experiment, all stimuli were performed on HKLs isolated from the same animal. For each independent experiment, each condition and each timepoint was tested in triplicate. Time points were based on preliminary gene expression studies (see supplemental figure S3). For gene expression analysis, *n* = 4 independent experiments (*n* = 4 fish) per species was performed.

### Total RNA isolation and cDNA synthesis

Total RNA from cells in RLT lysis buffer (Qiagen, 79216) was isolated using the RNeasy mini Kit (Qiagen, 74106), including on-column Dnase treatment, according to the manufacturer's instructions, and stored at −80 °C. Prior to cDNA synthesis, 750 ng RNA was treated with DNase I, Amplification Grade (Invitrogen), and cDNA was synthesized using random primers (300 ng, Invitrogen) and Superscript III First-Strand Synthesis for RT-PCR (Invitrogen). cDNA samples were diluted in nuclease-free water prior to real-time quantitative PCR (RT-qPCR) analysis.

### Gene expression analysis

Gene expression was measured with RT-qPCR using ABsolute qPCR SYBR Green Mix (Thermo Scientific) in a Rotor-Gene Q (Qiagen), and fluorescence data were analysed using Rotor-Gene Analysis software version 2.3.5. Briefly, 7 µL ABsolute qPCR SYBR Green Mix was combined with 2 µL primer mix (2.1 µM) and 5 uL cDNA and RT-qPCR was initiated by 15 min at 95 °C, subsequently 40 cycles of 20-second denaturation at 95 °C, 20 s annealing at 60 °C and 20 s extension at 72 °C followed by a stepwise melting curve with 0.25 °C per 5 s steps. The relative expression ratio (R) of each sample was calculated according to the Pfaffl method [34] based on the take-off deviation of each sample *versus* each of the PBS controls and normalized relative to *elongation factor 1α* (*elf1α*) as previously verified reference gene (see Tables 3 and 4 for the primers used).

**Table 3:** Overview of Nile tilapia RT-qPCR primers used in the current study. In case of (genome), sequences from related species were used as BLAST query against the GCA_001858045.3 genome build and primers were designed using the recovered rainbow trout sequences.

Primer	Forward (5′ – 3′)	Reverse (5′ – 3′)	Accession Code
*elf1α*	CCCACATCAACATCGTG	CCTTCAGTTTGTCCAGCA	AB075952
*il1b*	TAGTGCTGGGGGTAAAC	CACAGTGTCAACGCTT	DQ061114.1
*il10*	ACTCAGATGGAGAGCAG	CTGTGTCGTTTAGAAGCC	KP645180.1
*Tnfα*	TGGATATGGAAAGTGTGC	GCTCTGTTTTGTCGCC	AY428948.1
*Ifnγ*	CCAGCAGAGATGAACTTGA	CTCCAAAACACCACCCA	NM_001287402.1, KF294754.1
*il12p40*	ACTTTGGGTGAGCAGAAC	ATCCTCGCTGTGGCA	GCA_001858045.3 (genome)
*irak1*	CCTATGTTCCTGCTCCTG	GGTGTTCTGTATGGGTGTG	MF479259.1
*irak4*	CTGCCAATGTTCTGCTAC	GTTCGGTGTTCTCCTCT	MF479260.1
*myd88*	GTGTAAACGGATGGTGGT	GTAAAATGCTGGGGAAGG	KJ130039.1
*card9*	GTGTGAGAGGAGTGTGA	TGAAGAGTCATTCTGCTGC	XM_005464275.4
*bcl10*	CACTACCTCTGCGATAAAATC	TCTCAGCCAGTATGTCCA	XM_003437842.5

**Table 4:** Overview of rainbow trout RT-qPCR primers used in the current study. Primers were designed to discriminate between duplicated genes. All duplicated genes were tested individually, but only genes found regulated are presented. The last letter or number of the primer designates the duplicated gene. Further, * indicates the use of common primers (detecting duplicated genes). In case of (genome), sequences from related species were used as BLAST query against the GCA_013265735.3 genome build and primers were designed using the recovered rainbow trout sequences.

Primer	Forward (5′ – 3′)	Reverse (5′ – 3′)	Accession Code
elf1α*	TCTACAAAATCGGCGGTA	CCTCAGTGGTGACATTAGC	AF498320.1
*il1b2*	CTGGGTGCTCAACGAA	TAGTCTCACTGGGGACA	GCA_013265735.3 (genome)
*il10a*	CCCTGCTGGACGAA	TTGTTGTTCTGTGTTCTGT	GCA_013265735.3 (genome)
*tnfα2*	CGGAATGGAGCCTCAG	CTGTGTCAGCGGTAAGAT	AJ401377
*ifnγ2*	AGGGAGAGGCTGGAC	GGTGTCACTGGTCTTGAT	GCA_013265735.3 (genome)
*il12p40b*	CTACATCCGAGAAATAGTGAA	CACTTATAAACACCTTTTCCTT	HE798543.1
irak1*	GACAGTATCTCCGATGTGG	GCTTGTCCAGTACGTTCA	GCA_013265735.3 (genome)
irak4*	CTTCTTAGCGAGCGACT	CTTCACTCTGGGGTCC	GCA_013265735.3 (genome)
myd88*	TCAAGAATTACGAGGATTGC	TCAATGAGAACTCGGAGAT	GCA_013265735.3 (genome)
*card9-1*	TGTCCGCCAGCAC	CACTGTTCTTCTCTTCGC	GCA_013265735.3 (genome)
*bcl10a-1*	GGCAGTAGTGAAGCACG	TCCTGGTTTTGGCAGAC	GCA_013265735.3 (genome)

### Statistical analyses

Statistical analysis was performed using IBM SPSS statistical data editor version 26. Data presented as fold changes were transformed with natural logarithm, prior to statistical analyses. Subsequently, transformed data were tested for normality by using a Q-Q plot and performing a Shapiro-Wilk test. Data was then analysed using a repeated measures linear mixed model followed by a Bonferroni post hoc test. Analysis of gene expression differences was performed without testing for the factor ‘time post stimulation’ (differences between 3 h or 6 h), however this factor was taken into account as repeated measure. All values are means expressed with standard deviation (SD), and data were considered significant for *p*<0.05.

## Results

### Chemical analysis of MSP rich algae extracts reveals distinct differences between two species of algae and processing techniques

Algae were processed and, in total, eight different MSP-rich extracts were produced. Composition (proximal analysis) of the crude extracts differed (Table 5), not only based on the species that the extracts were derived from, green *Ulva* (UC) *versus* red *Solieria* (SC), but were also different based on the extraction methods. Differences were noted in the mineral and carbon content between the two crude processed extracts, most-likely attributable to the difference between the algae species. The osidic composition is not shown here but provided in Supplementary Table S1.

**Table 5:** Proximal analyses of the different MSP-rich extracts (% dry matter).

Extract	Dry matter	Mineral matter	Carbon	Nitrogen	Sulphur	Sugar	Protein	Uronic acid	Sulphate groups
UC	91.9	40.4	23.2	1.8	8.7	20.3	7.9	14.7	9.15
UC-T	97.9	32.6	28.8	2.7	7.2	29.4	24.6	13.3	15.4
UC-E	97.3	24.6	32.4	4.2	5.3	27.0	12.8	11.5	25.7
SC	93.1	64.4	14.9	2.9	6.6	11.9	11.6	1.2	4.2
SC-T	99.0	40.3	24.5	2.0	8.6	31.4	12.4	2.8	20.6
SC-E	96.0	28.8	29.6	3.2	5.3	36.1	16.2	1.5	6.1
UC-F15	95.4	47.3	20.6	1.4	5.7	9.3	8.1	8.7	0.6
UC-F50	91.1	58.8	17.9	1.3	8.8	11.0	8.2	3.6	1.3

For abbreviations see also Table 1. UC= *Ulva* concentrate untreated; UC-T = *Ulva* thermal extraction; UC-E = *Ulva* protease extraction; SC = *Solieria* concentrate untreated; SC-T = *Solieria* thermal extraction; SC-E = *Solieria* protease extraction; UC-F15 = *Ulva* filtrated at 15 kDa; UC-F50 = *Ulva* filtrated at 50 kDa.

### MSP-rich extracts from *Solieria chordalis* have more potent direct antimicrobial effects on pathogenic fish bacteria than MSP-rich extracts from *Ulva* sp

In order to analyse antimicrobial effects of the MSP-rich algae extracts, we cultured several common pathogenic fish bacteria and titrated the eight different MSP-rich extracts into the bacterial cultures. In general for each of the analysed MSP-rich extracts antibacterial effects were most pronounced at the earliest timepoint (24 h incubation or 72 h incubation for slow growing bacteria (*i.e. Aeromonas salmonicida, Aliivibrio salmonicida, Streptococcus agalactiae, Streptococcus iniae*)) and as such only those timepoints are presented in Table 6. Overall, extracts derived from red (*Solieria*) algae (SC, SC-T and SC-E) showed more pronounced antimicrobial effects than MSP-rich extracts isolated from green (*Ulva*) algae (Table 6). To extracts stood out in their antibacterial activity; SC-T and SC-E. For these two extracts, we observed clear antibacterial effects, at relatively low concentrations, on most of the tested bacterial species. Overall, we observed a certain degree of antibacterial effects for each of the MSP-rich extracts for at least one of the tested bacterial species.

**Table 6:** Analysis of antimicrobial effects of MSP-rich extracts against fish pathogenic bacteria. Values represent the dose (mg mL^−1^) at which there was inhibition of growth, as determined by OD600 and visual determination. A value of >12.5 mg mL^−1^ indicates absence of growth inhibition at this highest dose tested. Lower values indicate growth inhibition at the dose indicated. Bold and underlined values indicate growth inhibition at a dose <1.0 mg mL^−1^.

	UC	UC-T	UC-E	SC	SC-T	SC-E	UC-F15	UC-F50
**“Fast” growing bacteria**	mg mL^−1^			mg mL^−1^			mg mL^−1^	
*Aeromonas hydrophila*	>12.5	>12.5	>12.5	>12.5	1.56	1.56	6.25	6.25
*Edwardsiella tarda*	>12.5	3.13	**0.78**	3.13	**0.39**	**0.78**	>12.5	>12.5
*Edwardsiella ictaluri*	12.5	1.56	1.56	6.25	**0.20**	1.56	6.25	12.5
*V. anguillarum*	3.13	**0.78**	**0.78**	3.13	**0.78**	6.25	3.13	6.25
*Yersina ruckeri*	>12.5	1.56	1.56	6.25	**0.39**	3.13	12.5	>12.5
*Aeromonas veronii*	12.5	>12.5	>12.5	>12.5	6.25	**0.78**	3.13	6.25
*Shewanella putrefaciens*	3.13	3.13	1.56	1.56	6.25	**0.78**	6.25	1.56
*Vibrio vulnificus*	**0.78**	**0.78**	**0.78**	**0.78**	**0.78**	**0.39**	1.56	3.13
*Photobacterium damselae*	>12.5	**0.78**	1.56	**0.78**	**0.78**	**0.20**	**0.78**	>12.5
“Slow” growing bacteria	mg mL^−1^			mg mL^−1^			mg mL^−1^	
*Aeromonas salmonicida salmonicida*	6.25	3.13	3.13	>12.5	1.56	3.13	>12.5	>12.5
*Aliivibrio salmonicida*	>12.5	>12.5	>12.5	>12.5	>12.5	3.13	>12.5	>12.5
*Streptococcus agalactiae*	3.13	3.13	**0.78**	3.13	1.56	1.56	1.56	6.25
*Streptococcus iniae*	1.56	>12.5	**0.78**	>12.5	>12.5	6.25	**0.78**	3.13

Note: inhibition of bacterial growth after 24 h for ‘fast’ growing bacteria, or after 72 h for ‘slow’ growing bacteria. Data from later timepoints not shown.

For abbreviations see also Table 1. UC = *Ulva* concentrate untreated; UC-T = *Ulva* thermal extraction; UC-E = *Ulva* protease extraction; SC = *Solieria* concentrate untreated; SC-T = *Solieria* thermal extraction; SC-E = *Solieria* protease extraction; UC-F15 = *Ulva* filtrated at 15 kDa; UC-F50 = *Ulva* filtrated at 50 kDa.

### MSP-rich extracts induce ROS production in leukocytes from Nile tilapia and rainbow trout

**Fig. 1:**
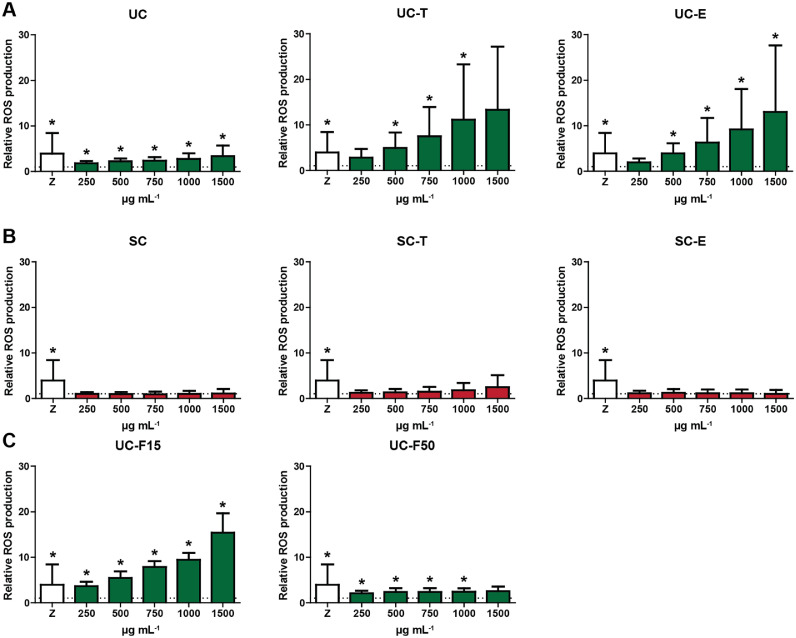
Induction of reactive oxygen species (ROS) in head kidney leukocytes (HKLs) of Nile tilapia after stimulation with MSP-rich algae extracts. HKLs were isolated and stimulated with zymosan (Z; 50 µg mL^−1^, open bars) as positive control, or with different concentrations of *Ulva* derived (1A), *Solieria* derived (1B) or filtered *Ulva* derived (1C) MSP extracts (250 - 1500 µg mL^−1^). Total ROS production was measured immediately following stimulation of cells for 2 h. Bars indicate mean + SD of *n* = 4 independent experiments. Asterisk (*) indicates significant difference (*p* < 0.05) relative to the corresponding control (dotted line, cells stimulated with culture medium only) as assessed by a linear mixed model, followed by an LSD post hoc test. Significant differences between concentrations are not indicated.

**Fig. 2:**
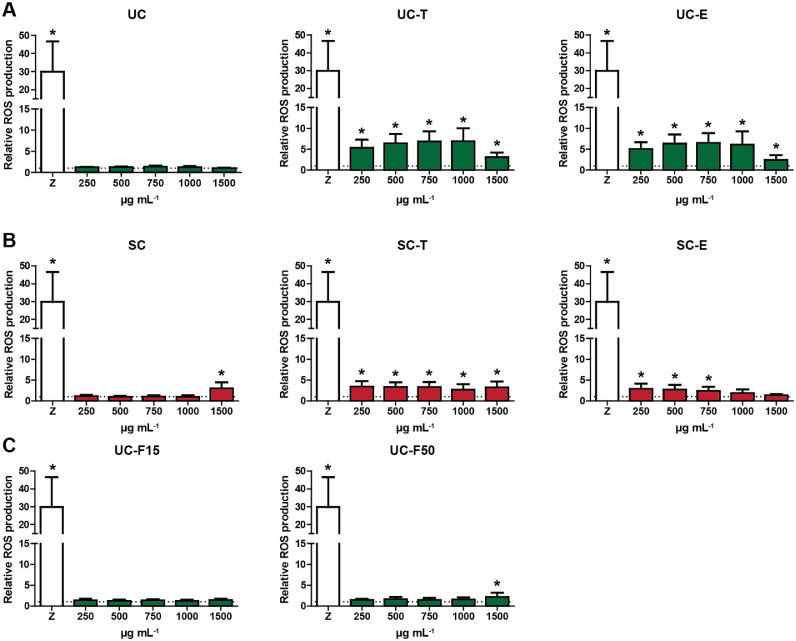
Induction of reactive oxygen species (ROS) in head kidney leukocytes (HKLs) of Rainbow trout after stimulation with algae extracts. HKLs were isolated and stimulated with zymosan (50 µg mL^−1^, open bars) as positive control, or with different concentrations of *Ulva* derived (1A), *Solieria* derived (1B) or filtered *Ulva* derived (1C) MSP extracts (250 - 1500 µg mL^−1^). Total ROS production was measured immediately following stimulation of cells for 2 h. Bars indicate mean + SD of *n* = 4 independent experiments. Asterisk (*) indicates significant difference (*p* < 0.05) relative to the corresponding control sample (dotted line, cells stimulated with RPMI only) as assessed by a linear mixed model, followed by an LSD post hoc test. Significant differences between concentrations are not indicated.

In HKLs isolated from Nile tilapia, MSP-rich extracts from green algae (*Ulva* sp.) significantly induced ROS production in a dose-dependent manner, albeit to different degrees, whereas none of the red algae *Solieria-*derived extracts induced ROS production significantly higher than the base line (RPMI) control (Fig. 1). Also in HKLs isolated from rainbow trout, MSP-rich extracts significantly induced ROS production. Responses in rainbow trout were different from responses in Nile tilapia, indicating fish species specific responsiveness, already clear from much higher relative ROS responses induced by the positive control zymosan in rainbow trout (Fig. 2).

Different from Nile tilapia, red algae *Solieria-*derived extracts did induce significant ROS production in HKLs isolated from rainbow trout, albeit without a clear dose-dependent responses. Similar to Nile tilapia, green algae *Ulva*-derived extracts also stimulated ROS production in HKLs of rainbow trout, however in a less consistent manner and without a clear dose-dependent response. ROS induction by MSP-rich extracts could be most reliably evaluated in Nile tilapia, with clear dose-responses for several green algae *Ulva*-derived extracts.

### MSP-rich extracts induce gene expression responses after stimulation *in vitro,* differing between Nile tilapia and rainbow trout HKLs

We employed the fact that HKLs from common carp, more than HKLs isolated from Nile tilapia and rainbow trout, easily produce NO *in vitro*, to titrate MSP-rich extracts prior to subsequent studies on gene expression analysis in Nile tilapia and rainbow trout. Thus, based on the titration of ROS induction in Nile tilapia and rainbow trout fish species, *and* based on the ROS and NO production in common carp (Supplementary Figures S1 and S2), we chose to proceed our studies with a fixed concentration of 500 µg mL^−1^ MSP-rich algae extract for gene expression analysis after stimulation. Genes studied were either cytokines, or genes associated with signalling pathways downstream of receptors sensing pathogen-associated molecular patterns (PAMPs), with a specific focus on the pathways associated with Toll-Like Receptors (TLR) and C-type Lectin Receptors (CLR). Gene expression was studied at two time points (3 and 6 h) after stimulation of HKLs.

**Fig. 3:**
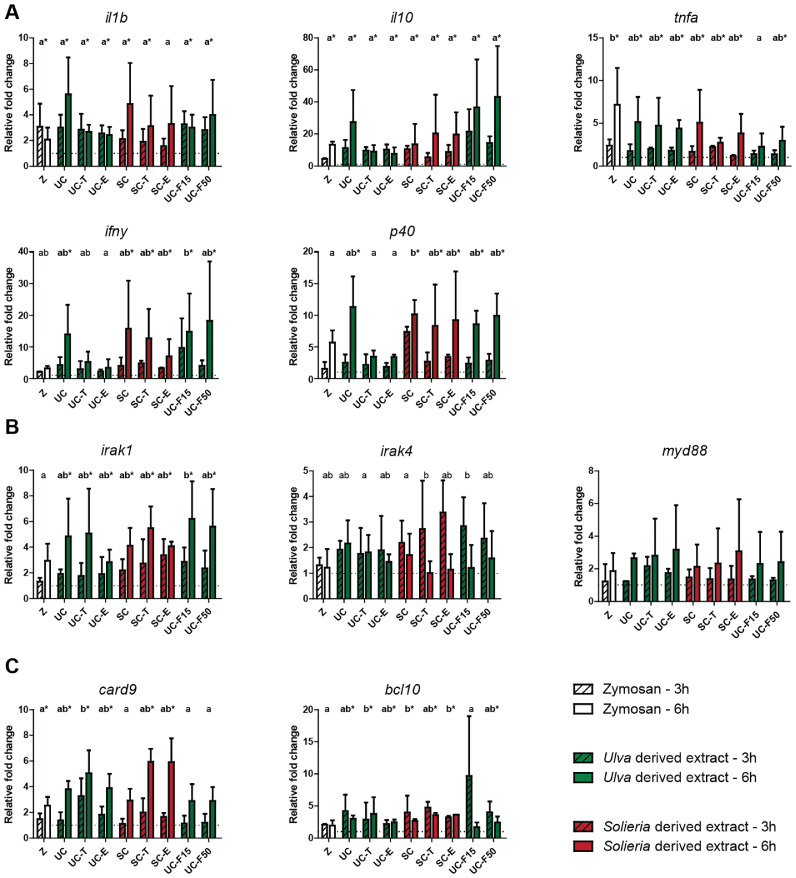
Effects of *Ulva* derived and *Solieria* derived MSP-rich extracts on gene expression of cytokines and selected receptor signalling molecules in Nile tilapia head kidney leukocytes (HKLs). Gene expression of selected cytokines (3A), TLR pathway associated signalling molecules (3B) and CLR associated signalling molecules (3C) after stimulation with zymosan (50 µg mL^−1^, white bars), *Ulva* sp. derived extracts (500 µg mL^−1^, green bars) or *Solieria* derived extracts (500 µg mL^−1^, red bars), at 3 h post stimulation (striped bars) or 6 h post stimulation (open bars). Gene expression is expressed relative to RPMI-stimulated HKLs (negative control, dotted line) and normalized for *elf1α* expression. All values are means expressed with standard deviation (SD) of *n* = 4 independent experiments, and data were considered significant for *p*<0.05. Significant differences between groups (overall regulation per treatment) were assessed by linear mixed model followed by Bonferroni post hoc test. Differences between time points post stimulation were not tested for significance. Groups with different letters are statistically different from one another, with ‘a’ indicating the lowest value. Groups with asterisk (*) are statistically different from the negative control.

**Fig. 4:**
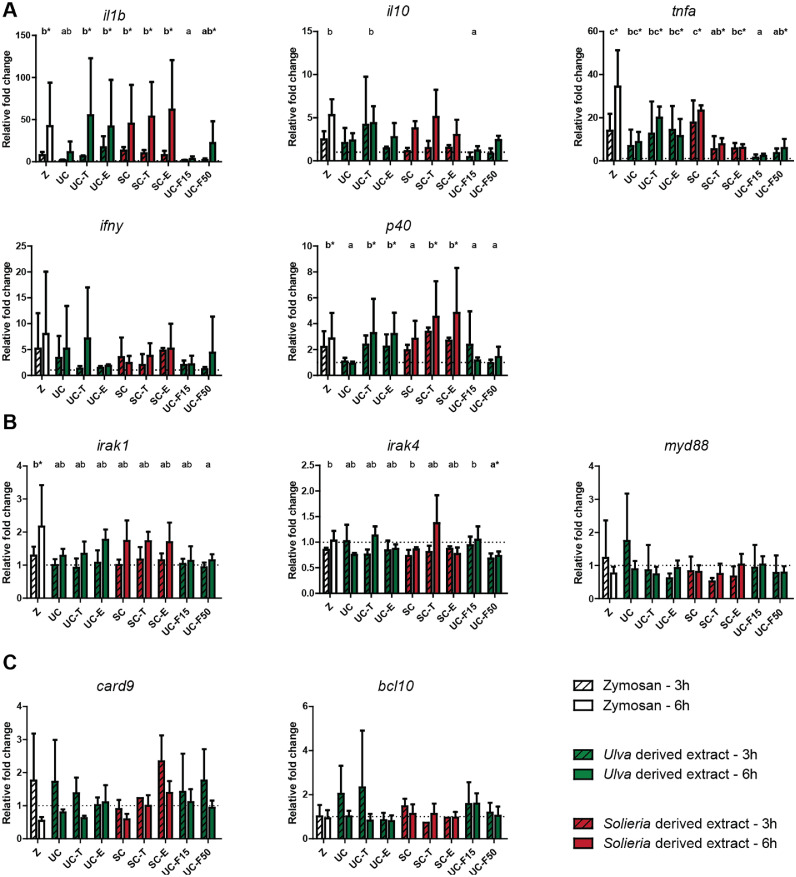
Effects of *Ulva* derived and *Soleiria* derived MSP-rich extracts on gene expression of cytokines and selected receptor signalling molecules in rainbow trout head kidney leukocytes (HKLs). Gene expression of selected cytokines (4A), TLR pathway associated signalling molecules (4B) and CLR associated signalling molecules (4C) after stimulation with zymosan (50 µg mL^−1^, white bars), *Ulva* sp. derived extracts (500 µg mL^−1^, green bars) or *Soleiria* derived extracts (500 µg mL^−1^, red bars), at 3 h post stimulation (striped bars) or 6 h post stimulation (solid bars). Gene expression is expressed relative to RPMI stimulated HKLs (negative control, dotted line) and normalized for *elf1α* expression. All values are means expressed with standard deviation (SD) of *n* = 4 independent experiments, and data were considered significant for *p*<0.05. Significant differences between groups (overall regulation per treatment) were assessed by linear mixed model followed by Bonferroni post hoc test. Differences between time points post stimulation were not tested for significance. Groups with different letters are statistically different from one another, with ‘a’ indicating the lowest value. Groups with asterisk (*) are statistically different from the negative control. Note: for rainbow trout gene expression paralogous genes showed comparable patterns and only one paralog is shown.

Analysis of cytokine gene expression revealed a clear induction of most of the studied cytokines (*interleukin-1β (il1b); interleukin-10 (il10); tumour necrosis factor-α (tnfa); interferon-γ (ifny); interleukin-12 p40 (p40)*). Induced cytokine responses generally were more prominent in Nile tilapia than in rainbow trout (*cf.* Figs. 3A and 4A). Although regulation of cytokine expression appeared ubiquitous, differences in gene expression of *il10* and *ifny* were less informative in rainbow trout. Despite the differences between the two fish species, cytokine read-outs suggested immunomodulatory effects of most if not all red algae *Solieria-*derived and green algae *Ulva*-derived extracts.

Analysis of gene expression associated with TLR signalling pathways (*irak1, irak4* and *myd88*) only revealed significant regulation of *irak1* and only in Nile tilapia (*cf.* Figs. 3B and 4B). Analysis of gene expression associated with CLR signalling pathways (*card9* and *bcl10*) revealed regulation of both, *card9* and *bcl10,* again only in Nile tilapia (Figs. 3C and 4C). Gene expression analysis suggested the immunomodulatory effects of MSP-rich extracts in Nile tilapia could possibly regulate at least CLR-mediated signalling, independent of algae source. Comparable to the use of ROS production as read-out for suggested immunomodulatory effects of red algae *Solieria-*derived and green algae *Ulva*-derived extracts, gene expression as read-out was dependent on the fish species investigated. Overall, expression of cytokine genes in particular, appeared most informative, especially for measuring immunomodulatory effects in Nile tilapia.

## Discussion

Increased interest in the use of dietary supplementation with immunomodulators such as marine sulphated polysaccharides (MSP), to maintain the health of animals in intensive aquaculture systems, has triggered a need for research on the underlying cell-based mechanisms of their modulatory capacities. MSP are highly complex sulphated polysaccharides extracted from cell wall matrices of marine algae, with their immunomodulatory mechanisms mostly detailed by studies in higher vertebrate species. So far, effects of MSP on fish cells, and also effects of MSP on fish pathogenic bacteria, had not been investigated. Here, we investigated the effects of (eight) different crude extracts rich in MSP from green macroalgae (*Ulva* sp.) or red macroalgae (*Solieria* sp.), first for direct antibacterial effects, and then for *in vitro* immunomodulatory action on primary head kidney leukocytes (HKLs) from Nile tilapia, and rainbow trout.

Direct antimicrobial effects of MSP-rich algae extracts were investigated by titration on cultures of 13 different bacterial strains representing both, Gram-positive and Gram-negative fish pathogens. Although to some degree we observed antibacterial effects for each of the MSP-rich extracts, extracts derived from red (*Solieria*) algae showed more pronounced antibacterial effects than MSP-rich extracts isolated from green (*Ulva*) algae. Inhibitory values were defined as concentrations at which there was notable inhibition of growth after a predetermined time period of 24–72 h, and could be as low as 0.20 mg mL−1. These values are comparable to the lowest minimum inhibitory concentration (MIC) of 0.16 mg mL−1 for MSP extracts prepared from green (*Ulva*) algae, when tested against 42 bacterial strains and isolates from livestock animals [12,17]. Although in the latter studies only green (*Ulva*) algae were included, our data suggest that antibacterial activities of MSP could be host species specific to some extent. Our study further suggests that algal species *per se* (here, *Solieria versus Ulva*) could be an important determinant, and that either thermal processing or protease assisted extraction appears beneficial for antibacterial properties of the MSP-rich extracts. Overall, MSP-rich extracts appear to have direct antibacterial effects, at least *in vitro*, and might be of use to combat bacterial infections or aid in maintaining microbiota homeostasis of both, livestock and aquaculture animals.

MSP-rich extracts derived from green *Ulva* algae stimulated ROS production by HKLs from both, Nile tilapia and rainbow trout. While the absolute ROS values differed per fish species (rainbow trout > Nile tilapia), a dose-dependent ROS production was most clearly detectable for Nile tilapia derived HKLs stimulated with *Ulva*-derived extracts. Overall, ROS production by HKLs was less obvious for MSP-rich extracts derived from red *Solieria* algae, compared to MSP-rich extracts derived from green *Ulva* algae. At first sight, this outcome favouring MSP extracted from green *Ulva* algae appears in contrast with the above-discussed outcome favouring MSP extracted from red *Solieria* algae for their anti-bacterial activities. The mechanisms involved in direct antibacterial effects, however may be very different from the mechanisms inducing ROS production by HKLs, and the exact determinants present in MSP-rich extracts may not necessarily be represented in equal amounts in extracts from red *Solieria* algae and extracts from green (*Ulva*) algae. In other words, antibacterial killing and ROS production by HKLs, are functional read-outs differing to such an extent that they provide very different arguments for selecting the ‘best’ MSP extract from either red (*Solieria*) algae, or green (*Ulva*) algae.

Pathogen-associated molecular patterns such as likely present in MSP-rich extracts could be sensed by pattern recognition receptors (PRR) from the TLR family, but could also be sensed by other PRR such as those from the C-type lectin receptors (CLR) family. In human embryonic kidney (HEK293) cells, MSP-rich extracts activated cells primarily through Toll-Like Receptor 4, with subsequent activation of signalling via the transcription factor NF-κB, leading to the secretion of several cytokines [12]. In the absence of reporter assays for PRR-driven activation of fish cells, analysis of induced expression of genes associated with particular PRRs and their downstream signalling pathways might provide useful information. Here, in Nile tilapia, gene expression analysis of *irak1, irak4* and *myd88* after stimulation of HKLs with MSP-rich extracts showed regulation of *irak1* only. At the same time, gene expression analysis of *card9* and *bcl10* showed regulation of both genes, suggesting that the recognition of MSP-rich extracts in HKLs might be mediated by a member of the CLR family or that MSP-rich extracts might induce the CLR signalling pathway. Of course, cellular reporter assays should be useful to confirm these preliminary observations on the sensing of MSP by particular cellular receptors on fish cells. Analysis of cytokine gene expression in MSP-stimulated HKLs revealed a clear induction of most of the cytokines studied (*il1b, il10, tnfa, ifny, il-12 p40*). Since cytokine gene expression can be the result of many different signalling pathways, this provides no further information on a PRR responsible for sensing MSP-rich extracts. Regardless of the exact receptor and downstream signalling pathway involved, the increased gene expression of several cytokines shows the immunomodulatory properties of MSP-rich extracts in Nile tilapia and rainbow trout, at least *in vitro*.

Gene expression data from Nile tilapia showed, in general, more obvious results, compared to data from rainbow trout. Further, data from Nile tilapia showed, in general, more pronounced effects, compared to data from rainbow trout when it came to studying ROS production by HKLs. In addition, data from common carp were again different from Nile tilapia and rainbow trout; HKLs isolated from carp reacted to almost all MSP-rich extracts with both, enhanced ROS production and nitric oxide (NO) response (supplementary data). Given that Nile Tilapia are *Cichlidae*, common carp are *Cyprinidae*, and rainbow trout are *Salmonidae*, biological diversity between members of such different fish families should not come as a surprise. It may be evident that investigations into the immunomodulatory effects of MSP-rich extracts based on effects found in a single species cannot always be simply extrapolated to several target fish species with different physiology.

Chemical analysis of the different MSP-rich extracts showed clear differences in the composition related to the different algal species but also in the effects of the different treatments. Of interest, sugar content of extracts increased due to either thermal extraction or protease-assisted extraction compared to crude cold-press extraction. For *Ulva-*derived extracts a similar treatment effect was observed for sulphate groups content, however, this was not as evident for *Solieria-*derived extracts. Revisiting the ROS and gene expression analyses with the chemical composition of the different extracts in mind, we found no clear relation between the potency of an induced ROS or gene expression response and the chemical composition of the extracts. This implies that observed immunomodulatory effects of the tested *Ulva-* and *Solieria-*derived extracts might not solely depend on the presence or absence of certain sulphate groups or polysaccharide content, but may also depend on other properties, for instance of structural nature.

Finally, for future experiments on immunomodulatory effects of MSP-rich extracts from either red (*Solieria*) or green (*Ulva*) algae, one could have a small preference for using green (*Ulva*) algae extracts. For Nile tilapia, none of the red (*Solieria*) algae*-*derived extracts induced ROS production by HKL at levels significantly higher than the base line control, while MSP-rich extracts derived from green (*Ulva*) algae induced ROS in a dose-dependent manner. Cytokine gene expression read-outs suggested immunomodulatory effects of most if not all MSP-rich extracts from either red (*Solieria*)*-* or green (*Ulva*) algae, so provide no information on preference for which algae extract to use, not for Nile tilapia nor for rainbow trout. For common carp, MSP-rich extracts derived from green (*Ulva*) algae induced ROS production more so than MSP-rich extracts from red (*Solieria*) algae, although products from both, red (*Solieria*)*-* and green (*Ulva*) algae triggered NO production.

With regard to processing, it could be of interest to study *in vivo*, differences, or the lack of differences, induced by either cold extraction or thermal processing, and investigate correlations with higher concentrations of active material, or differences in composition. For future experiments with immunostimulatory MSP-rich extracts from green (*Ulva*) algae, one could have a small preference for using UC-T treatments; *i.e.*2 h of thermal processing at 80 °C to extract immunostimulatory MSP-rich extracts. In our hands, treatment effects differed between assays and between fish species and thus were not unidirectional, but thermal processing is a relatively reliable process that could be preferred by suppliers. Preferably, future experiments on the immunomodulatory effects of MSP-rich extracts could focus on thermally-processed extracts from green (*Ulva*) algae and address *in vivo* feeding, respecting biological differences between target species.

## Declaration of Competing Interest

The authors declare the following financial interests/personal relationships which may be considered as potential competing interests: Jules Petit reports financial support was provided by Ministry of Agriculture Nature and Food Quality.

## Data Availability

Data will be made available on request.

## Appendix Supplementary materials

Image, application 1
