# Supplementary material for: Green and red macroalgae extracts show antibacterial effects and induce innate immune responses in Nile tilapia and rainbow trout in vitro
Source: Comp Immunol Rep. 2023 Dec 22;6:200128. doi: 10.1016/j.cirep.2023.200128 (PMC10865728; doi:10.1016/j.cirep.2023.200128)
Supplement: Supplementary file 1 [file mmc1.docx]

# Supplementary data

Supplementary Table S1: Osidic composition of the different MSP-rich extracts. Gal refers to galactose, Glc refers to glucose, Xyl refers to xylose, Man refers to mannose, Rha refers to rhamnose, GlcA refers to glucuronic acid and IduA refers to iduronic acid.

|  | Osidic composition in mass ratio | | | | | | | Osidic composition in molar ratio | | | | | | |
| --- | --- | --- | --- | --- | --- | --- | --- | --- | --- | --- | --- | --- | --- | --- |
| Extract | **Gal** | **Glc** | **Xyl** | **Man** | **Rha** | **GlcA** | **IduA** | **Gal** | **Glc** | **Xyl** | **Man** | **Rha** | **GlcA** | **IduA** |
| UC | 0.98 | 1.1 | 1.66 | 0.44 | 8.01 | 6.99 | 3.3 | 0.1 | 0.11 | 0.21 | 0.05 | 1 | 0.67 | 0.32 |
| UC-T | 4.94 | 2.16 | 2.44 | 0.53 | 6.55 | 5.5 | 2.49 | 0.63 | 0.27 | 0.37 | 0.07 | 1 | 0.65 | 0.29 |
| UC-E | 4.33 | 2.25 | 2.13 | 0.47 | 5.93 | 4.66 | 1.98 | 0.57 | 0.3 | 0.34 | 0.06 | 1 | 0.55 | 0.24 |
| SC | 4.39 | 0.69 | 5.08 | *ND* | *ND* | *ND* | *ND* | 1 | 0.16 | *ND* | *ND* | *ND* | *ND* | *ND* |
| SC-T | 10.46 | 4.86 | 0.59 | *ND* | *ND* | *ND* | *ND* | 1 | 0.46 | 0.07 | *ND* | *ND* | *ND* | *ND* |
| SC-E | 12.53 | 7.36 | 0.63 | *ND* | *ND* | *ND* | *ND* | 1 | 0.59 | 0.06 | *ND* | *ND* | *ND* | *ND* |
| UC-F15 | 3.24 | 0.57 | 3.51 | 1.54 | 29.26 | 12.49 | 11.95 | 0.09 | 0.02 | 0.12 | 0.04 | 1 | 0.33 | 0.32 |
| UC-F50 | *ND* | *ND* | *ND* | *ND* | *ND* | *ND* | *ND* | *ND* | *ND* | *ND* | *ND* | *ND* | *ND* | *ND* |

ND = not determined

## Common carp head kidney leukocyte isolation

European Common carp (*Cyprinus carpio carpio* L.) originated from a cross between the Hungarian R8 strain and the Polish R3 strain (Irnazarow, 1995). Carp were reared at 21 ± 2 °C temperature with a 12-12h light-dark cycle in the Aquatic Research Facilities of Wageningen University and Research (Carus-ARF, Wageningen, The Netherlands). Fish were fed a commercial diet (Skretting) twice per day. Carp were euthanized with 0.3 g L^-1^ tricaine methanesulphonate in aquarium water buffered with 0.6 g L^-1^ sodium bicarbonate, and bled via the caudal vein. Head kidneys were removed aseptically and total head kidney leukocytes (HKLs) were isolated as previously described (Pietretti et al., 2013).

## Induction of reactive oxygen species (ROS) production by MSP-rich extracts

Production of ROS was determined by a real-time luminol-based luminescence assay, as previously described with minor modifications (Petit et al., 2021). Briefly, HKLs were seeded at a density of 1x10^6^ per well and incubated for 60 minutes at 27°C in white 96-well plates (CLS3912; Corning). Subsequently, cells were stimulated with one of the following: RPMI cell culture medium (negative control), zymosan (positive control; tlrl-zyd, 50 mg ml^-1^, InvivoGen) or one of the extracts (Table 1) UC, UC-T, UC-E, SC, SC-T, SC-E, UC-F15 or UC-F50 at a concentration of 250, 500, 750, 1000 and 1500 µg mL^-1^. Chemiluminescence emission was measured in real time (every 2 min for 120 min) with a FilterMax F5 Multi-Mode Microplate Reader at 27˚C, and expressed as fold changes based on areas under the curve (Petit et al., 2019). Fold changes were based on data from stimulated relative to unstimulated HKLs (treated with RPMI).

## Induction of nitric oxide (NO) production by MSP-rich extracts in common carp

Production of NO was determined as nitrite accumulation using the Griess reaction, as previously described (Saeij et al., 2002). HKLs were seeded at a density of 1x10^6^ per well in 96-well culture plates (CORN3596; Corning) and stimulated with the one of the following: RPMI cell culture medium (negative control), zymosan (positive control; tlrl-zyd, 50 mg mL^-1^, InvivoGen) or one of the extracts (Table 1) UC, UC-T, UC-E, SC, SC-T, SC-E, UC-F15 or UC-F50 at a concentration of 50, 100, 250, 500 and 750 µg mL^-1^. After 96h at 27˚C in the presence of 5% CO_2_, nitrite production was measured at OD540, using a FilterMax F5 Multi-Mode Microplate Reader and quantified using a sodium nitrite (NaNO_2_) standard curve.

**References**

Irnazarow, I. 1995. Genetic variability of Polish and Hungarian carp lines. Aquaculture, 129(1-4), 215-215.

Petit, J., Embregts, C.W.E., Forlenza, M., Wiegertjes, G.F. 2019. Evidence of Trained Immunity in a Fish: Conserved Features in Carp Macrophages. J Immunol, 203(1), 216-224.

Petit, J., Wiegertjes, G.F. 2016. Long-lived effects of administering beta-glucans: Indications for trained immunity in fish. Dev Comp Immunol, 64, 93-102.

Pietretti, D., Vera-Jimenez, N., Hoole, D., Wiegertjes, G. 2013. Oxidative burst and nitric oxide responses in carp macrophages induced by zymosan, MacroGard® and selective dectin-1 agonists suggest recognition by multiple pattern recognition receptors. Fish & shellfish immunology, 35(3), 847-857.

Saeij, J.P., Van Muiswinkel, W.B., Groeneveld, A., Wiegertjes, G.F. 2002. Immune modulation by fish kinetoplastid parasites: a role for nitric oxide. Parasitology, 124(Pt 1), 77-86.

Supplementary Figure S1: Induction of reactive oxygen species (ROS) in head kidney leukocytes (HKLs) of common carp after stimulation with MSP-rich algae extracts. HKLs were isolated and stimulated with zymosan (Z; 50 µg mL^-1^, open bars) as positive controls, or with different concentrations of *Ulva* derived (A), *Solieria* derived (B) or filtered *Ulva* derived (C) MSP extracts (250 - 1500 µg mL^-1^). Total ROS production was measured immediately following stimulation of cells for 2 hours. Bars indicate mean + SD of n=4 independent experiments. Asterisk (*) indicates significant difference (*p* < 0.05) relative to the corresponding control sample (dotted line, cells stimulated with culture medium only) as assessed by a linear mixed model, followed by an LSD post hoc test. Significant differences between concentrations are not indicated.


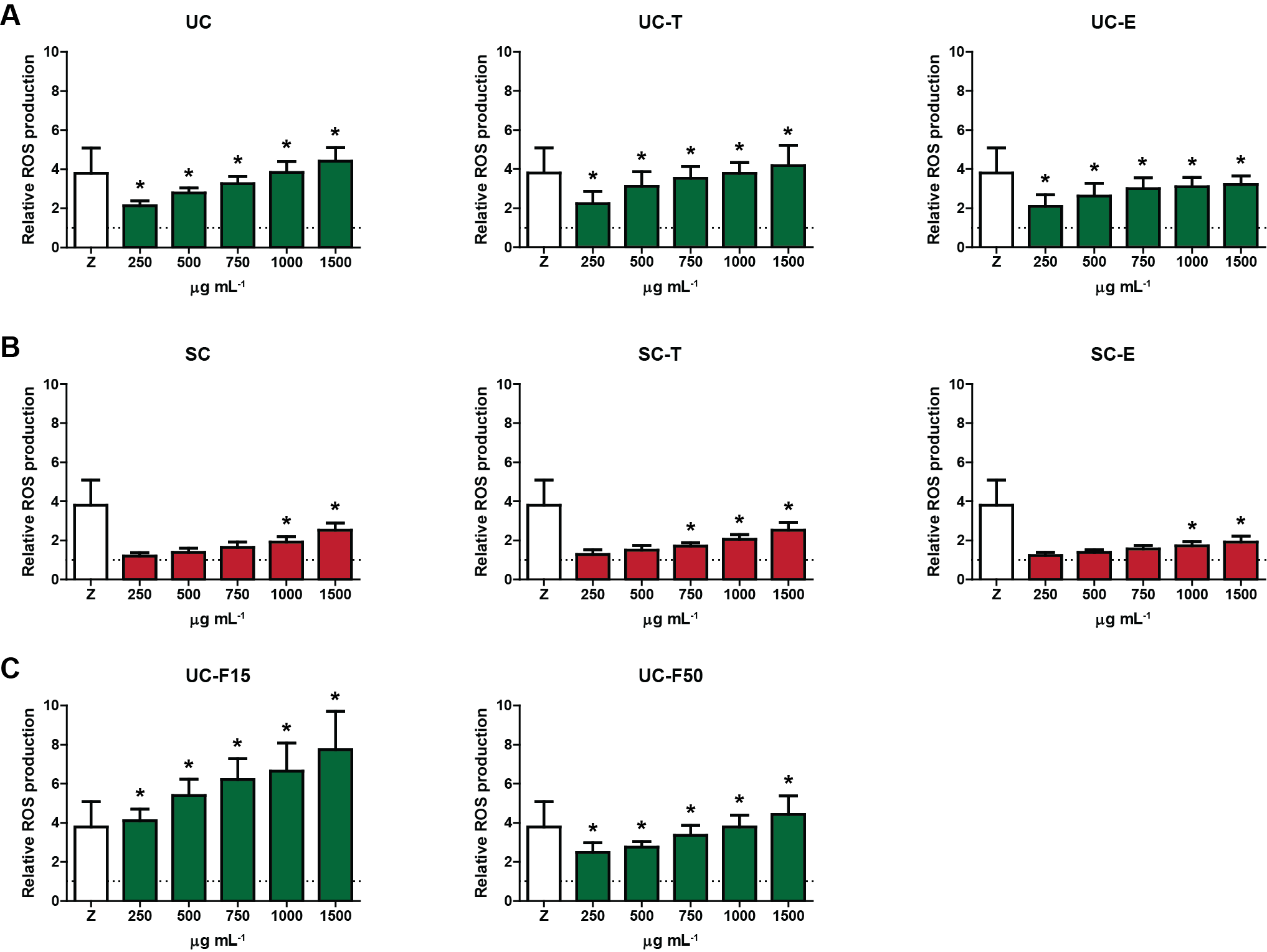


Supplementary Figure S2: Induction of nitric oxide production by head kidney leukcoytes (HKLs) of common carp after stimulation with MSP-rich algae extracts. HKLs were isolated and stimulated with zymosan (Z; 50 µg mL^-1^, open bars) as positive controls, or with different concentrations of *Ulva* derived (A), *Solieria* derived (B) or filtered *Ulva* derived (C) MSP extracts (50 - 750 µg mL^-1^). After 96h incubation nitric oxide (NO) accumulation in the supernatant was analysed. Bars indicate mean + SD of n=5 independent experiments. Asterisk (*) indicates significant difference (*p* < 0.05) relative to the corresponding control sample (dotted line, cell stimulated with culture medium only) as assessed by a linear mixed model, followed by an LSD post hoc test. Significant differences between concentrations are not indicated.


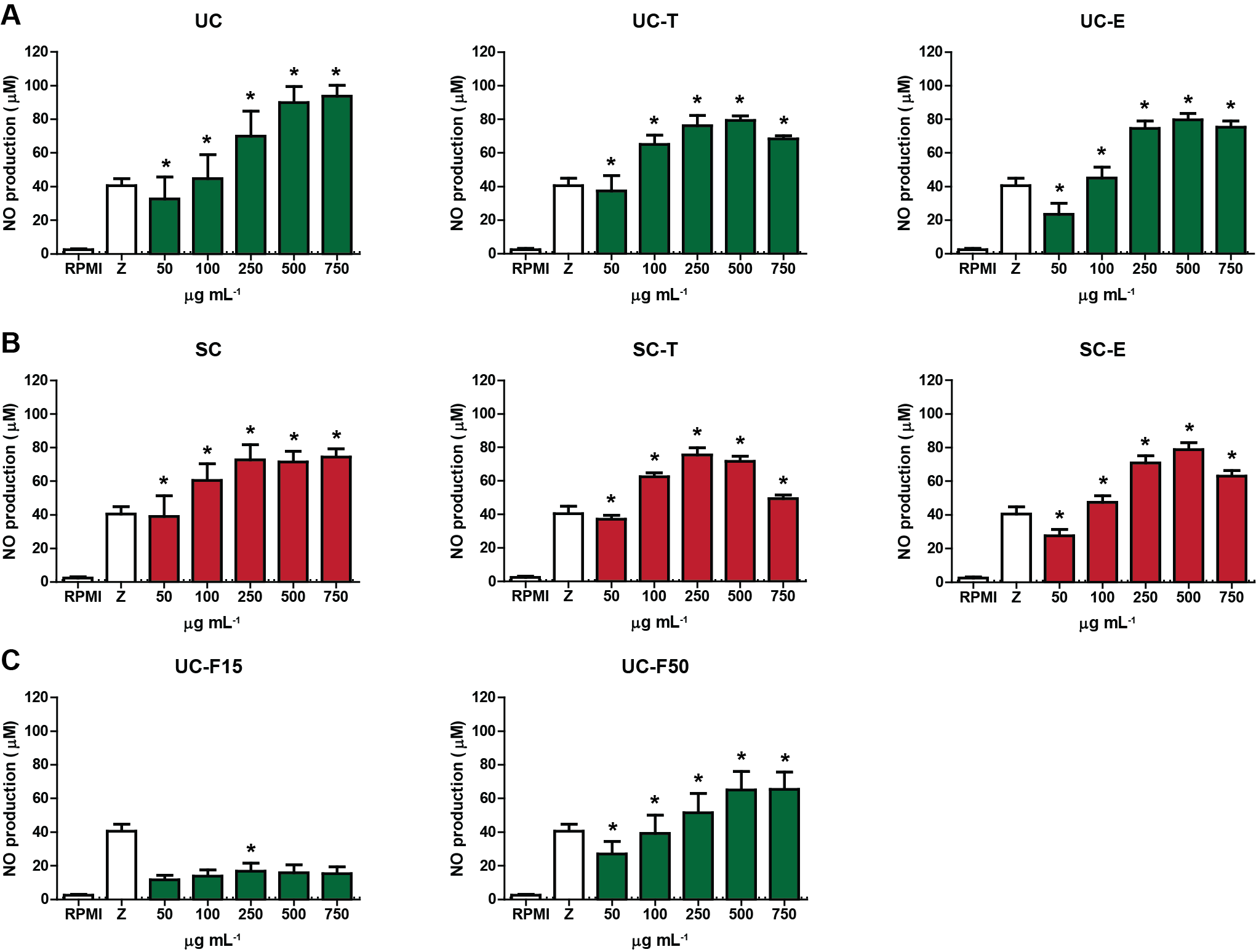

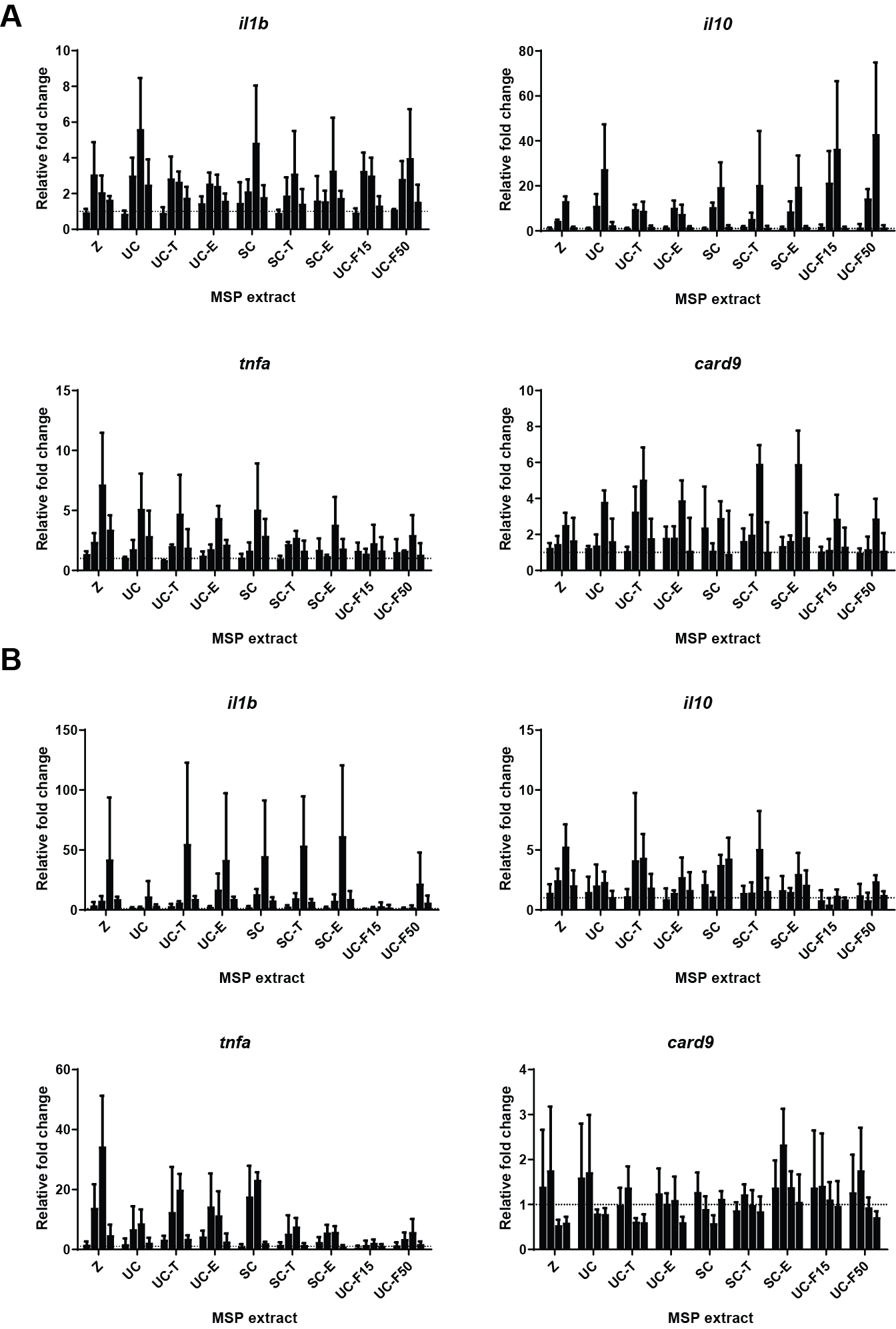


Supplementary Figure S3: Kinetics of gene expression regulation in head kidney leukocytes (HKLs) of Nile tilapia (A) and rainbow trout (B). Gene expression of selected genes (pro-inflammatory, anti-inflammatory and transcription factor) after stimulation with zymosan (50 µg mL^-1^) or each of the MSP rich extracts (500 µg mL^-1^) at 1, 3, 6 and 15 hours post stimulation. Gene expression is expressed relative to RPMI-stimulated HKLs (negative control, dotted line) and normalized for *elf1α* expression. Bars express mean + SD of n=2 independent experiments (1 and 15 hours post stimulation) or of n=4 independent experiments (3 and 6 hours post stimulation). Four bars per treatment express relative fold change at the consecutive time points (1, 3, 6 and 15 hours post stimulation).
